# A new primary dental care service compared with standard care for child and family to reduce the re-occurrence of childhood dental caries (Dental RECUR): study protocol for a randomised controlled trial

**DOI:** 10.1186/s13063-015-1010-9

**Published:** 2015-11-04

**Authors:** Cynthia Pine, Pauline Adair, Girvan Burnside, Louise Robinson, Rhiannon Tudor Edwards, Sondos Albadri, Morag Curnow, Marjan Ghahreman, Mary Henderson, Clare Malies, Ferranti Wong, Vanessa Muirhead, Sally Weston-Price, Hilary Whitehead

**Affiliations:** Institute of Dentistry, Barts and The London School of Medicine and Dentistry, Queen Mary University of London, Turner Street, Whitechapel, London E1 2BA UK; R&D Department, Salford Royal NHS Foundation Trust, Mayo Building, 3rd Floor, Stott Lane, Salford, M6 8HD UK; Health Psychology and Behavioural Medicine Research Group, School of Psychological Sciences and Health, University of Strathclyde, 40 George Street, Glasgow, G1 1QE UK; Department of Biostatistics, University of Liverpool, Institute of Translational Medicine, Crown Street, Liverpool, L69 3BX UK; Centre for Health Economics and Medicines Evaluation, Ardudwy Hall, Bangor University, Bangor, Gwynedd LL57 2PZ UK; School of Dentistry, University of Liverpool, Pembroke Place, Liverpool, L3 5PS UK; NHS Tayside, Tweed Place, Perth, PH1 1TJ UK; North East London NHS Foundation Trust, Tantallon House, Goodmayes Hospital Site, Barley lane, Essex, IG3 8XJ UK; Kent Community Health NHS Trust, Capital House, Jubillee Way, Faversham, Kent ME 13 8GD UK; Salford Royal NHS Foundation Trust, Community Dental Service, Dental Department, Pendleton Gateway, 1 Broadwalk, Salford, M6 5FX UK

**Keywords:** Caries prevention, Children, Post-extraction, Dental nurse-led, Brief negotiated interview, Cost-effectiveness

## Abstract

**Background:**

In England and Scotland, dental extraction is the single highest cause of planned admission to the hospital for children under 11 years. Traditional dental services have had limited success in reducing this disease burden. Interventions based on motivational interviewing have been shown to impact positively dental health behaviours and could facilitate the prevention of re-occurrence of dental caries in this high-risk population. The objective of the study is to evaluate whether a new, dental nurse-led service, delivered using a brief negotiated interview based on motivational interviewing, is a more cost-effective service than treatment as usual, in reducing the re-occurrence of dental decay in young children with previous dental extractions.

**Methods/Design:**

This 2-year, two-arm, multicentre, randomised controlled trial will include 224 child participants, initially aged 5 to 7 years, who are scheduled to have one or more primary teeth extracted for dental caries under general anaesthesia (GA), relative analgesia (RA: inhalation sedation) or local anaesthesia (LA). The trial will be conducted in University Dental Hospitals, Secondary Care Centres or other providers of dental extraction services across the United Kingdom. The intervention will include a brief negotiated interview (based on the principles of motivational interviewing) delivered between enrolment and 6 weeks post-extraction, followed by directed prevention in primary dental care. Participants will be followed up for 2 years. The main outcome measure will be the dental caries experienced by 2 years post-enrolment at the level of dentine involvement on any tooth in either dentition, which had been caries-free at the baseline assessment.

**Discussion:**

The participants are a hard-to-reach group in which secondary prevention is a challenge. Lack of engagement with dental care makes the children and their families scheduled for extraction particularly difficult to recruit to an RCT. Variations in service delivery between sites have also added to the challenges in implementing the Dental RECUR protocol during the recruitment phase.

**Trial registration:**

ISRCTN24958829 (date of registration: 27 September 2013), Current protocol version: 5.0.

**Electronic supplementary material:**

The online version of this article (doi:10.1186/s13063-015-1010-9) contains supplementary material, which is available to authorized users.

## Background

Tooth decay (dental caries) is a preventable disease; children are at increased risk because newly erupted teeth are more susceptible [1]. Caries occurs following consumption of frequent sugary foods and drinks combined with the lack of a counterbalance of regular fluoride, for example, through twice daily tooth brushing with fluoridated toothpaste [2]. Prevalence of childhood caries is related to material deprivation with the highest levels of disease generally being present in the most deprived communities [3, 4]. The Northwest of England has some of the highest levels of caries experienced in the United Kingdom, with, for example, 47 % of 5-year-old children in Salford affected [4]. In Scotland, although the level of dental caries has decreased in recent years, approximately 25 % of the children still experience caries in primary dentition [5].

In young children, most caries are untreated, with the most common dental treatment being extraction of the primary teeth [6]. Extractions in general dental practice are carried out under local anaesthesia or sedation, whereas in the hospital, general anaesthesia (GA) is commonly used. In England and Scotland, dental extraction is the single highest cause of planned admission to the hospital for children under 11 years of age [7]. One in 10 children in the United Kingdom has experienced tooth extraction by age 5, with 5 % of children having extractions under GA [8]. Tooth extraction can be traumatic for a child, particularly when performed under GA, and while these are costly interventions, they can also generate life-long adult dental anxiety [9].

In 2013/14, 46,500 children and young people under 19 were admitted to hospital for a primary diagnosis of dental caries; in the 5- to 9-year-old age group, dental caries was the most common reason for children to be admitted to hospital [7]. Children having primary teeth extracted are significantly more likely to develop further decay and have their first permanent molars extracted. A new dental service that can reduce the re-occurrence of dental decay has the potential to result in reductions in re-attendance in hospital for tooth extraction, reduction of dental pain and lost days from school, improvements in future child oral health and reduction in oral sepsis.

Following national guidance on dental GA, dentists have become more radical in the number of primary teeth extracted under a single GA in order to reduce the incidence of repeat GA when carious teeth have been left behind [10, 11]. However, despite addressing the burden of caries experience in the deciduous dentition and reducing repeat GA, surgical treatment is not effective in preventing further tooth decay because the underlying aetiological factors remain, and these are usually behavioural, such as a lack of twice daily tooth-brushing with a fluoride toothpaste, uncontrolled dietary sugars and irregular dental attendance [12]. Therefore, there may be no reduction in decay experienced in children’s permanent teeth. A study of children having first permanent molar teeth extracted in the Northwest of England found 70 % were due to caries, 40 % had undergone previous extractions (of primary teeth), and 68 % had no previous dental treatment for the decayed permanent teeth [13]. There are associations between children with severe tooth decay requiring extractions and being underweight, perhaps reflecting poor diets [14, 15], with NICE guidance recognising dental neglect as a potential indicator of general neglect [16].

Children with caries are more likely to attend the dentist only when they have symptoms such as pain, and their parents are more likely to be dentally anxious and not attend the dentist regularly [17]. Establishing preventive dental care post-extraction is challenging because many families are from poorer backgrounds and do not have a routine of regular dental attendance; for example, one study found that of those who failed to attend the dentist, 83 % were from deprived areas, with only 17 % from more affluent areas [18]. Parents’ lack of engagement with dental care following extractions in their children is found in other countries [19]. The importance of family environment [20] and broader social determinants of health are thus critical to understand barriers to healthy behaviours [21].

Educational interventions for changing tooth-brushing and sugar intake behaviours are on their own ineffective [22]. Interventions that take account of parental attitudes and beliefs towards children’s oral health behaviours have a greater chance to be effective because previous research has indicated that these factors predict dental caries in young children [23]. Establishing healthy behaviours in young children can lead to significant savings in dental care, enhanced well-being and quality of life [24]. Multi-country studies have found that parents of children in low socio-economic groups have lower parental self-efficacy (PSE) in relation to dental health-related behaviours and are less confident in establishing routines [23, 25]. This correlation has been mirrored in other studies whereby a link between parental self-efficacy and better oral health outcomes for children has been indicated [19, 26].

In addition to lower PSE, parents whose children subsequently develop new decay post-extraction have been found to be less receptive to advice, more permissive to their child’s desires and at earlier stages of change [27]. Readiness to change is an important predictor of whether parents adopt prevention [28]. New approaches need to be applied that address these factors and have been shown to be effective in other chronic disease management. Developing tailored individualized feedback based on participants’ risk behaviours appropriate to readiness to change may result in significant long-term health behaviour change and health improvement [19].

### Motivational interviewing for improving health

Intervention studies based on motivational approaches have demonstrated that they can move people from inaction to action and that this is an effective approach, which has demonstrated efficacy in lifestyle behaviours such as smoking [29]. It is a particularly useful approach when resistant (for example, non-adherence to oral health advice) behaviours are present. No UK dental studies using a brief negotiated intervention based on motivational interviewing for preventative oral health care have been reported. However, US researchers have developed successful interventions influencing parents to adopt and maintain dental preventive behaviours for their children [1, 30, 31]. These studies established a relation between measures of parenting beliefs and behaviours and change status and the presence/absence of dental disease [32]. Using a motivational interviewing (MI) approach positively impacted dental behaviours, with fewer carious lesions 1 year after a single MI intervention [31]. Extending the length of interventions and intensive follow-up after MI may enhance efficacy further [33]. A range of healthcare staff can deliver MI interventions following appropriate training. Traditionally however, such training is intensive because the targeted behaviours are often addictive (for example, substance abuse) and interventions have been delivered within specialist services [34]. MI has been adapted for delivery as a brief intervention in a medical setting. This model delivers MI in 30 minutes or less using a structured and clear framework taught to practitioners in a brief training programme [35]. While this approach is useful to raise awareness as a starting point for behavioural change, Emmons and Rollnick point out that supplementing this with printed materials and ongoing support may be necessary for longer term behavioural change. This approach has been shown to be of patient benefit in non-dental conditions and in changing negative attitudes, beliefs and behaviours. Following pilot work and based on the literature, we have developed a psycho-social intervention, the Dental Recur Brief Negotiated Interview (DR-BNI), which was designed to be delivered to parents of children who have had a dental extraction. DR-BNI may offer significant benefits for patients because it works in collaboration with them, developing empathy and a shared understanding of their situation regarding their child’s oral health. This takes place while taking into account personally formulated arguments for and against change, particularly through facilitating conversations that support reasons for changing behaviour (change talk) and decreasing talk about maintaining the status quo or staying the same (sustain talk) [36].

The aim of this randomised controlled trial is to test the efficacy and cost-effectiveness of an ASDN (Additional Skills Dental Nurse)-delivered intervention of the Dental Recur Brief Negotiated Interview for Oral Health (DR-BNI). The focus of the intervention is on increasing parental self-efficacy for three oral health-related behaviours aimed at reducing the re-occurrence of dental caries in children who have previously had a primary tooth extracted.

### Aims

This research aims to develop and evaluate a new dental service (DR-BNI), which has been designed with representative families through partnership working with Children’s Centres. It re-orientates the service away from symptomatic to preventive care to be tailored to patients and their families’ needs and preferences in accessing dental care.

The primary aim of the trial is to evaluate whether a new dental nurse-led service, delivering a brief negotiated interview, is a more cost-effective service than that currently offered in the United Kingdom by dentists in reducing the re-occurrence of dental decay in young children with previous dental extractions. Secondary outcome measures include parental self-efficacy (PSE) to undertake dental health-related behaviours for their child, consumption of sugary foods and drinks and tooth-brushing behaviours, parental attitudes to child dental health, and parental values of dental health-related behaviours.

## Methods/Design

This is a two-arm, multi-centre randomised controlled trial (RCT), with blinded outcome assessment.

### Ethics, consent and permissions

Research ethics and governance approval has been gained for this study through the IRAS system from Greater Manchester Central NRES committee (REC ID: 13/NW/0466) and Salford Royal Foundation Trust R & D Department. Local NHS permissions (R&D approval) for individual sites and participating GDPs have been obtained from the Clinical Research Network Greater Manchester, The Royal Liverpool and Broadgreen University Hospitals NHS Trust, Clinical Research Network - North West Coast, Tayside Medical Science Centre, RM&G Consortium for Kent & Medway, Clinical Research Network North Thames and North East London NHS Foundation Trust.

### Study sample and recruitment

Participants will be identified from University Dental Hospital extraction clinics, Secondary Care Centres or other providers of dental extraction services or extraction clinics in deprived areas across England and Scotland. The parents/legal guardians of patients, aged 5 to 7 years, who are scheduled to have one or more primary teeth extracted for dental caries under general anaesthesia (GA), relative analgesia (RA; inhalation sedation) or local anaesthesia (LA) will be advised by the clinic dental nurse or dentist that a trial is being conducted in the centre and that their child and family are eligible to be considered for recruitment. At the trial end, the children will be 7 to 9 years. It is anticipated that should new caries develop, the majority will be on the first permanent molars. Therefore, children will be excluded if they are scheduled to have all of their first permanent molar teeth extracted. Other exclusions are currently participating in any other trial or having done so in the previous 3 months or being classified as severely disabled.

Parents and guardians will be given sufficient time to consider the study and opportunity to ask questions before informed consent is taken. Informed consent will be obtained for each participant.

In the United Kingdom, all patients are asked to attend an assessment appointment prior to their extraction, and recruitment will take place either at the assessment appointment, a pre-extraction appointment or subsequent extraction appointment depending on preference of the Centre.

Parents of children who choose to participate in the study will receive the intervention either whilst attending a routine appointment (that is, at assessment, pre-extraction or extraction) or where this is not possible, at an additional mutually convenient appointment between enrolment and the 6-week post-operative period. The intervention will be conducted by the ASDN. Participants will be sent a reminder by text or telephone the day before the intervention appointment where appropriate.

All general dental practices in the study areas will be advised by letter that the trial is commencing. The study design will be described as will the practice role should one of their patients consent to join. Each practitioner will be given the option to advise the research team if they prefer for their practice be excluded.

### Randomisation

Randomisation of participants will occur just prior to any intervention being delivered. Parents who do not attend for the intervention will be judged to have not accepted the invitation to join the trial and will be sent a letter thanking them for their initial interest and advising them that they have not been entered into the trial.

Randomisation will use block randomisation stratified by recruiting centre, with random variable block sizes. Randomisation sequences will be generated by a statistician at the Liverpool Clinical Trials Research Centre (CTRC), who is not part of the trial statistical team. The trial statistical team will be blinded to allocation until the final statistical analysis plan has been completed and agreed upon.

Each centre will be supplied with a set of pressure sealed sequentially numbered randomisation envelopes. Randomisation of participants will be carried out by a trained member of clinic staff opening the next envelope in sequence, witnessed by a colleague. Group allocation will be to Group 1: DR-BNI or Group 2: Dental Development (control intervention). Parents or guardians will be informed of their group allocation.

### Interventions

Group allocation will determine the content of the intervention. Delivery of the interventions will be conducted by a trained Additional Skills Dental Nurse (ASDN). Both interventions, DR-BNI and Dental Development, are conducted as interviews with a similar structure of six sections of 5 minutes duration, flowing from one to the next. These have not been scripted to allow individual tailoring to the participant.

All interventions will be audio recorded using a digital recorder to support assessment of the fidelity of delivery of the intervention and facilitate supervised practice throughout the trial. Parents who attend for the intervention will receive a £5 gift voucher as a contribution to their expenses [37].

### The Dental RECUR Brief Negotiated Interview

Participants randomised to Group 1 will receive the DR-BNI.

ASDNs will attend training in delivering the DR-BNI; the training will be delivered by an experienced clinical and health psychologist (PA). The training programme will follow motivational interviewing principles combined with health behaviour change techniques [38] for promoting oral health. The ASDNs will be trained in change talk, developing a change plan, consolidating commitment and MI and other behavioural-change approaches relevant to promoting good oral health (tooth-brushing, sugar control and regular dental attendance).

The DR-BNI will be delivered as a 30-minute consultation of six segments (Build Rapport, Ask about Pros and Cons, Feedback, Readiness to change, Action Plan, Dental Appointment and thanks). The session will establish child dental risk behaviours and the readiness to change these behaviours. This will be followed by motivating the parent to make the necessary changes to improve their child’s oral health by respectful and collaborative communication with the parent. The aim is to explore opportunities with the parent that might lead to change in past behaviours rather than telling them what to do. The session will allow a tailored programme to be planned in relation to enhancing parental efficacy in three main child dental health-related behaviours: establishing twice daily tooth-brushing with fluoridated toothpaste, particularly at night-time; reducing the frequency of sugary foods and drinks; and attending for preventive rather than symptomatic dental care. The ADSNs are advised to try, if appropriate, and agree on two goals with the participant using the behaviours described in the modified dental contemplation ladder [39] (Additional file 1).

The agreed-upon goals will vary for each family and be a personal preventive goal, committing to establishing a specific dental health-related behaviour for their child, for example, changing from sugar-containing soft drinks to sugar-free and brushing their child’s teeth at bedtime with fluoridated toothpaste. At the end of the interview, each participant will be offered support to attend a general dental practice for regular, preventive care for their child over the next year.

### Placebo control

Participants allocated to Group 2 (Dental Development) will receive the control intervention. This intervention has the same structure as the DR-BNI, but the delivery mode is educational rather than negotiated goal setting. The ASDN will provide information and discuss subsequent dental development that occurs between 6 years and 14 years, the period when most of the children’s permanent teeth will erupt. The information will be structured around concepts of growing up, shedding and growing new teeth, and descriptions and illustrations of the eruption of permanent teeth with primary precursors followed by eruption of molars with no precursors. No discussion on prevention will occur within this control intervention session. Participants in the control group will not be disadvantaged because it is anticipated that prevention discussions will occur within their usual care.

All families (both DR-BNI and Placebo Control) will receive the same summary leaflet on dental development information to take home. At the end of the intervention, participants in the control group will be advised to continue with their child’s dental practice arrangements as usual.

### Post-intervention care for the Dental Recur Brief Negotiated Interview group

At the end of the intervention for DR-BNI families, the ASDN will assist the parent to make the first of their child’s quarterly recall appointments with their general dental practitioner (GDP). This should be within 3 months of the intervention. If the participant does not have a GDP, or their GDP has declined to participate in the study, the ASDN will assist the participant in finding a local practice and making their first appointment. The date of the first appointment will be noted and a reminder will be sent to the participant on the week of their appointment.

The GDPs of all participants will have been informed of their patients’ involvement in the research. The GDPs of participants allocated to the test group will also be notified of the goals agreed during the DR-BNI and will be sent advice regarding frequency of recall visits and preventive care as advised by the Department of Health (DH) in England in Delivering Better Oral Health [12] (a guidance manual on recommended prevention provided to each GDP in England by DH); GDPs in Scotland will be provided with a similar guidance document [5]. This guidance recommends that child patients at high risk for caries be recalled every three months. Children who have had a primary tooth extracted for dental caries are considered at high risk of caries in the future. As noted above, the first recall appointment will be arranged at the end of the DR-BNI intervention. All subsequent quarterly recall appointments will be organised between the participant and their GDP.

We will ask the test group participant’s GDP to complete a case report form (CRF) and send it back to the Co-ordinating Centre each time the participant attends their Dental Practice in the first year. In the second year, the practice will be asked to recall the child as advised by national guidelines (every 3 months if the child continues to be high risk). At the end of the second year we will contact the GDP to request details of appointments attended and those the patient failed to attend and any preventive advice or treatment. We may also access electronic payment records to obtain this information.

Following the intervention, participants randomized to the control group will be advised to take their child to their family GDP as normal. We will contact control group participants’ GDPs at 1 and 2 years (+/−3 months) post-enrolment to request details of appointments attended and those the patient failed to attend and any preventive advice or treatment. We may also access electronic payment records to obtain this information.

The GDPs will receive additional payment from research funds as a contribution to the extra costs of completing the research forms and facilitating follow up questionnaire completion by parents.

### Data collection

#### Primary outcome

The primary outcome will be dental caries experienced in the 2 years post-enrolment on any tooth in either dentition at the dentinal level of involvement, which had been caries free at baseline.

Following informed consent, the dentist at the assessment or extraction clinic will conduct a baseline dental assessment and complete a full dental charting including presence of caries in remaining teeth, presence of dental plaque on upper anterior teeth and include a note of teeth extracted/to be extracted. Dentists making the caries assessment are all experienced paediatric dentists who will have received standardised training from the PI on the level of dental caries to record, that is, lesions that involve the dentine.

At 2 years post-enrolment, dental officers, trained in standardised techniques for the measurement of dental caries, will examine participating children in their School or at a location convenient to the family. The dental officers will be blind to group allocation. Dental caries experience, at the dentinal level of involvement, on any tooth in either dentition will be recorded. All children will be examined using sterilised or single-use mouth mirrors, a standardized halogen lamp (2,000 lux) and cotton wool rolls as needed.

### Secondary outcomes

Families in both groups will be asked to complete a set of three questionnaires at baseline. Parental readiness to change and beliefs about caring for their children’s teeth will be measured by the modified Contemplation Ladder [39]. Parental self-efficacy in relation to the implementation of dental health-related behaviours for the study children will be measured by the Child Oral Health Behaviours Questionnaire [23] and Parenting Self-Efficacy Scale [40] will also be administered. The parental self-efficacy measures [22, 38] will be administered again at 1 year and 2 years post-enrolment. Use of dental services and child oral health behaviours, including dietary behaviours, also will be obtained at baseline, at 1 year and at 2 years post-enrolment [23]. Oral cleanliness will be measured by plaque assessment on the buccal surfaces of upper anterior teeth at dental examinations.

### Health economic analysis

Cost will be measured from a public sector, multi-agency perspective [41–44]. We will fully cost the community-based DR-BNI and prevention in dental practice programme (as compared with costs of usual dental care); record study participant dental service use, primary and secondary care health service use, social care and special educational service use (using a CSRI, (costed using National unit costs); conduct a primary cost-effectiveness analysis (using dental caries rates as our measure of effectiveness); and conduct a secondary cost-consequences study relating costs to a range of consequences spanning measures of dental decay in the participating child, regular dental attendance, parent participation in better oral health behaviours (for example, sugar-free bedtime routine) and school attendance.

### Sample size

The primary outcome variable is measured by a binary variable, taking the value 1 where a child has caries experience after 2 years on any tooth in either dentition, which was caries free at baseline, and 0 otherwise. In a previous clinical trial conducted by two of the investigators in children from deprived areas in Scotland [45], 55 children aged 5 years had extractions for caries at baseline. Of these, 48 (87 %) had additional caries experience 2 years later. Taking this value as an estimate of the outcome in the control group, and setting the minimum clinically significant difference to 20 percentage points (67 % in the test group), with 80 % power and significance level 0.05 gives a minimum sample size of 78 per group. Previous studies and the pilots suggest allowing up to 30 % for dropouts; final sample size becomes 112 children per group.

### Measures

The Oral Health Behaviours Questionnaire (OHBQ) has been developed in a multi-cultural international study to explore parental attitudes and behaviours in relation to child tooth-brushing, use of dietary sugar, dental attendance and to measure parental self-efficacy in relation to tooth-brushing and dietary sugar [23].

The Contemplation Ladder measures readiness to change behaviour (28). The ladder has been modified for this study to address specifically the four recommended behaviours: 1) brush child’s teeth last thing at night and on one other occasion every day, 2) make regular visits to dentist, 3) limit sugar snacks to mealtimes and no more than four times a day, and 4) the ideal drink for children is milk or water. All of these recommendations come from Delivering Better Oral Health [12].

A parental self-efficacy scale will enable the researchers to measure the impact of the intervention on both general parental self-efficacy (29) and parental self-efficacy related to oral health behaviours, which has been used in a previous study with good reliability [23].

### Method of analysis

All statistical analysis plans will be prepared by the trial statistician, and approved by the Chief Investigator, supervising statistician and Trial Steering Group (TSG) before the allocations are released to the trial statistician and comparative data analysis is carried out. The analysis of the primary outcome variable will use logistic regression, adjusted for the stratification variable centre, and possibly other pre-specified variables judged to be potentially related to the outcome. The primary outcome will be analysed on an intention-to-treat (ITT) basis. In addition, a per-protocol analysis will be carried out to test the robustness of the main results to departures from ITT. Protocol deviations will be defined prior to development of the statistical analysis plan. All participants will have any protocol deviations listed and signed off by the Chief Investigator prior to the release of the randomisation codes to the trial statistical team. Sensitivity analyses will be carried out using multiple imputation to investigate the robustness of the analysis to missing primary outcome data.

Secondary outcome variables will be analysed using linear regression for numerical variables, and logistic regression for binary categorical variables, adjusted for centre and pre-specified potential confounders, as in the primary outcome analysis.

## Discussion

To our knowledge, no previous randomised controlled trials have been published investigating prevention of dental caries in children who have previously had an extraction in their primary dentition. Those delivering the intervention are dental nurses, reflecting moves to skill mix in dental care, which is, to date, a rare feature in dental trials. The participants are a hard to reach group in which secondary prevention is a challenge. Children scheduled for extractions are less likely to have been taken to attend their dentist for regular preventive treatment. This lack of engagement with dental care makes the children and families scheduled for extraction particularly difficult to recruit to an RCT. Variations in service delivery between sites have also added to the challenges in implementing the Dental RECUR protocol during the recruitment phase.

The protocol has been refined to allow randomisation and intervention delivery to occur at any appointment after enrolment and up to 6 weeks post-extraction. Participants can therefore participate on the trial whilst attending their routine appointments or have the option to schedule an additional appointment if preferred. This design removes some of the barriers to participation associated with attendance at additional appointments, for example travel and parents’ needing to take time off work. The flexibility of the design also allows staff working on the trial to fit trial requirements around existing appointment schedules at their site.

## Trial status

This trial is currently recruiting participants.

## Additional file

Additional file 1:
**Modified dental contemplation ladder.** (DOCX 91 kb)

## Abbreviations

ASDN: Additional Skills Dental Nurse
CRF: case report form
DR-BNI: Dental RECUR Brief Negotiated Interview
GA: general anaesthesia
GDP: General Dental Practitioner
LA: local anaesthesia
ITT: intention to treat
MI: motivational interviewing
PSE: parental self-efficacy
RA: relative analgesia
TSG: Trial Steering Group
