# Supplementary material for: A new primary dental care service compared with standard care for child and family to reduce the re-occurrence of childhood dental caries (Dental RECUR): study protocol for a randomised controlled trial
Source: Trials. 2015 Nov 4;16:505. doi: 10.1186/s13063-015-1010-9 (PMC4634579; doi:10.1186/s13063-015-1010-9)
Supplement: Additional file 1: — Modified dental contemplation ladder. (DOCX 91 kb) [file 13063_2015_1010_MOESM1_ESM.docx]

**Additional file 1 - Modified dental contemplation ladder**

**
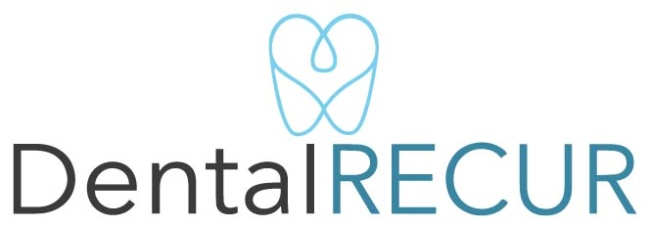
**

**Thinking about your child’s teeth and taking your child to the dentist regularly**

Each rung on the ladder represents where various parents are in how they think about the health of their child’s teeth. For each one please **Circle** the number that shows where **you** **are right now** in relation to the stated recommendation.

Prescription for Change:

**1) Brush child’s teeth last thing at night and on one other occasion every day (please circle where you are right now)**
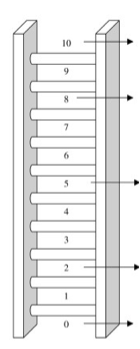


What steps do you need to take to change your current position (if required) – what would help you move up the ladder?

I have no thoughts about doing this at all

I think I need to consider doing this at some point but need help

I think I should be doing this but I struggle and am not quite ready

I am starting to think about how I can do this every day

I already do this every day

**
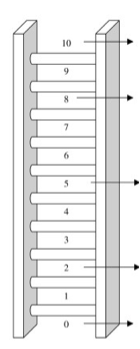
2) Take child regularly to the dentist (please circle where you are right now)**

What steps do you need to take to change your current position (if required) – what would help you move up the ladder?

I have no thoughts about doing this

I think I need to consider doing this at some point but need help

I think I should be doing this but I struggle and am not quite ready

I am starting to think about how I can do this

I already do this

**3) Control child’s sugar snacks to mealtimes and no more than 4 times in a day (please circle where you are right now)**


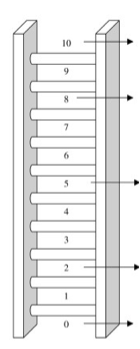


What steps do you need to take to change your current position (if required) – what would help you move up the ladder?

I have no thoughts about doing this

I think I need to consider doing this at some point but need help

I think I should be doing this but I struggle and am not quite ready

I am starting to think about how I can do this every day

I already do this every day

**4) Control child’s drinks to milk or water (please circle where you are right now)**


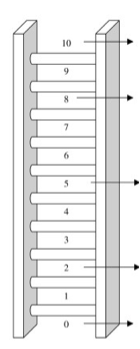


What steps do you need to take to change your current position (if required) – what would help you move up the ladder?

I have no thoughts about doing this

I think I need to consider doing this at some point but need help

I think I should be doing this but I struggle and am not quite ready

I am starting to think about how I can do this every day

I already do this every day
